# Consumer e-health education in HIV/AIDS: a pilot study of a web-based video workshop

**DOI:** 10.1186/1472-6947-6-10

**Published:** 2006-02-27

**Authors:** Laura A O'Grady

**Affiliations:** 1 Ontario Institute for Studies in Education of the University of Toronto, Toronto, Canada

## Abstract

**Background:**

Members of the HIV/AIDS community are known to use web-based tools to support learning about treatment issues. Initial research indicated components such as message forums or web-based documentation were effectively used by persons with HIV/AIDS. Video has also shown promise as a technology to aid consumer health education. However, no research has been published thus far investigating the impact of web-based environments combining these components in an educational workshop format.

**Methods:**

In this qualitative study HIV/AIDS community members provided feedback on an integrated web-based consumer health education environment. Participants were recruited through organizations that serve the HIV/AIDS community located in Toronto, Canada. Demographics, data on Internet use, including messages exchanged in the study environment were collected. A group interview provided feedback on usability of the study environment, preferences for information formats, use of the message forum, and other sources for learning about treatment information.

**Results:**

In this pilot study analysis of the posted messages did not demonstrate use for learning of the workshop content. Participants did not generally find the environment of value for learning about treatment information. However, participants did share how they were meeting these needs. It was indicated that a combination of resources are being used to find and discuss treatment information, including in-person sources.

**Conclusion:**

More research on the ways in which treatment information needs are being met by HIV/AIDS community members and how technology fits in this process is necessary before investing large amounts of money into web-based interventions. Although this study had a limited number of participants, the findings were unexpected and, therefore, of interest to those who intend to implement online consumer health education initiatives or interventions.

## Background

### HIV/AIDS community and use of the Internet

Due to the complex nature of HIV/AIDS and ever changing treatment options, many people with HIV/AIDS (PHAs) have taken the lead in their own health care, actively pursuing treatment information on their accord. Some have even created their own Internet web sites to help share information. Boberg and others [1] suggested that PHAs have specific needs including contact with other PHAs, access to social supports to aid decisions around treatment, and access to information about various social services available. It has also been argued that the complexity of HIV infection requires that PHAs have 'informational' support in dealing with these issues [1]. In addition, some initial research with PHAs who use the Internet for health care indicated they experience improved knowledge about the illness, increased skills in coping, and support from others [2]. Further, some believe the Internet is likely to become the main source of information for HIV/AIDS treatment and prevention information [3].

### Computer- Mediated Communication (CMC) in consumer health education

Various software applications such as e-mail, mailing lists, newsgroups, or web-based message boards are currently used for shared communication on the Internet. These tools have been employed to discuss a wide variety of issues, ranging from hobbies to work-related needs. Many individuals informally created online groups to specifically discuss shared health care issues [4]. People whose illness makes travel too difficult have used the Internet as a means to contact others in similar situations. For those seeking privacy, the ability to participate in virtual anonymity can also provide motivation to obtain health care information online [5]. HIV positive study participants have reported using this technology to connect with others [6]. Further, online content is available any time of the day or night from the privacy of home.

Some literature has been published regarding online support groups designed specifically for HIV/AIDS community members. An earlier software application CHESS (Comprehensive Health Enhancement Support System) used a bulletin board system (also known as BBS) technology. Unlike the Internet, a BBS requires a direct connection using telephone lines with a modem. It is a self-contained environment and, therefore, not as readily accessible as an Internet web site. The CHESS project originally developed a variety of modules, including ones for breast cancer and Adult Children of Alcoholics [7]. One component was also developed for HIV/AIDS. It contained treatment-related materials and also provided the means for people with HIV/AIDS (PHAs) to connect with others. Research on this initiative reported the Discussion Group (an area for posting messages to exchange information) was a very popular area for PHAs [1]. Another study focusing on HIV/AIDS using CHESS observed improvements in quality of life amongst participants [8]. However, a few disadvantages of online support groups have also been reported. Without the benefit of a professional moderator, groups run by laypersons may lead to erroneous information being shared. Also problematic in online communications in general is the lack of visual and social cues that can inhibit communication [9]. Off-topic posts and too many messages can also be a drawback.

### Multimedia and video in consumer health care education

Various publications also support the claim that multimedia technology can be used to accommodate learning, particularly for health care. For example, in an application of evidence-based approaches to health promotion, Robinson, Patrick, Eng and Gustafson [10] recommended using a variety of information modes including visual, audio, and text. They further suggest that access to professionals (experts) be provided for patrons of health care web sites. Rice [11] argued that, "Interactive media can improve health promotion because of increased learning, information seeking, information processing, and individualized knowledge by current or potential patients or interested parties." (p. 28).

Video has been used to assist consumers in making health care treatment decisions, describe what to expect when having a procedure done, and provide instruction on self care [12]. The advantages of using video for this purpose include that medical information is provided in a consistent manner [13], and patients can review the material at their convenience, starting and stopping the video when necessary [14]. Videos can also aid patients in the process of formulating questions they may have about their treatment [12]. To the author's knowledge, no research has been published in which video has been integrated into an online environment to aid consumer-oriented health care learning.

### Theoretical framework

Recent research in learning theories has focused on a socio-cognitive approach, one in which the learner constructs understanding through support derived from experience and participation in a group of other learners [15]. One publication [15] in instructional design (the development of educational materials to support learning) provides an approach to fostering a web-based learning environment. Recommended components to support learning include presentation/lecture, knowledge, and communication areas. The presentation/lecture area would contain text, video, and links to web sites with other content. The knowledge area would include general help references about accessing the web site. The communication area allows for message exchange [15]. A key aspect in supporting group learning is the ability to exchange messages within the interface.

### Study purpose

Although some research has been conducted in online distance education [16], little is known about how health consumers learn from web-based material presented outside formal educational settings. According to Reeves [6], "Yet despite the promise of online health resources, few studies have looked at how they are used by individuals coping with health problems, including HIV." (p. 48). Therefore, this research was designed to investigate how a web-based resource that included CMC, video, and support documents was used by HIV/AIDS community members. To operationalize this objective, the software application ePresence that combines these components, was implemented. More details on this application are provided in the Methods section. An accompanying web site containing support materials was also provided. As an exploratory endeavour, the intention was to investigate in what ways these various components (video, slide show, message forum, and accompanying web site) were used by study participants to learn.

## Methods

### Study environment

The topic area chosen for the workshop presentation was complementary and alternative health care (CAHC) as PHAs use these types of treatments frequently [17]. A presentation by an expert in this field, which was approximately twenty-five minutes in length, was video taped for use in the ePresence interface. In the presentation a brief overview was provided on Ayurveda (the science of life), nutrition, and yoga. All of these concepts were discussed in relation to HIV/AIDS. This was accompanied by a slide presentation, which provided an outline and bullet points of the material presented. These components (known collectively as the workshop) were integrated into the ePresence environment, which contained a message forum for exchanging information. The workshop used in this study was archived so study participants could view and review it at their convenience.

**Figure 1:**
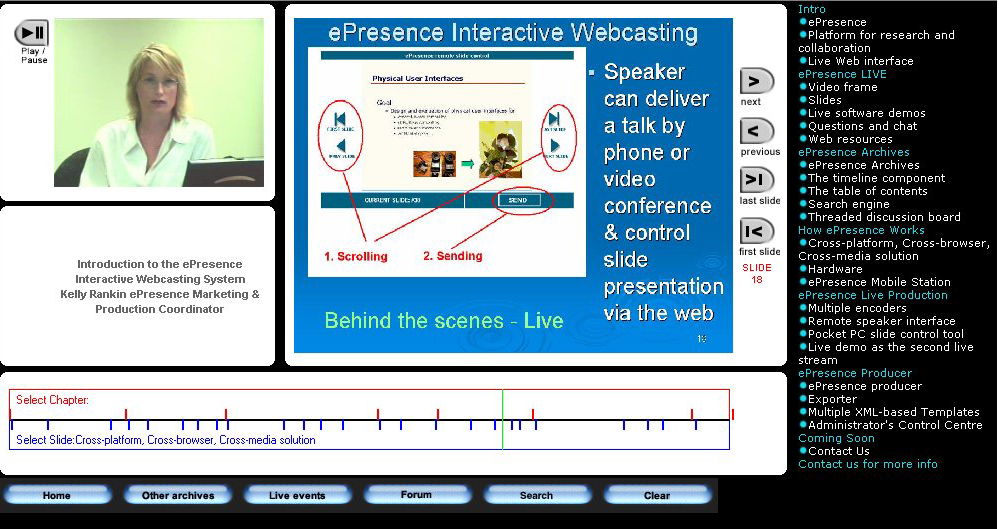
Image of ePresence Interactive Media Software. represents a screen image of the version of the ePresence software used in this research study. The woman depicted in the image is Kelly Rankin, ePresence Open Source Consortium Media Business Manager, who granted permission for her image to be used in this publication.

The ePresence interface is divided into four areas (Figure 1). The top left component streams a video presentation. The middle component renders a slide show. Located to the right of the slide show is an interactive table of contents. Clickable links allow the user to advance the slides as well as select individuals slides based on the title. Under these three main areas is an interactive mechanism allowing the user to choose specific points in the video presentation (represented by red coloured lines labelled "Select Chapters") and the slide presentations (represented by the blue coloured lines labelled "Select Slide"). Found across the bottom are a variety of links, including one to the message forum. An accompanying web site containing other support documents on CAHC in HIV/AIDS was also provided. The workshop, message forum and web site, known collectively as the study environment, were available to participants at all times.

Respondents were required to read and acknowledge an online consent form before participation. They were also asked to sign an identical informed consent at the in-person group interview. This informed consent included a request that all information shared in the meeting be kept confidential. Access to the study environment used in the research investigation was password protected. A separate username and password was also required to access the message forum within ePresence.

### Recruitment

Due to the specialized nature of the study participants were purposively recruited by advertising in settings where people with HIV/AIDS were known to frequent for services [18]. The study took place in downtown Toronto, Canada. It was advertised through local AIDS Service Organizations and other community-based associations by placing a poster about the study on bulletin boards accessible to the public. This poster requested participation from those who had an interest in learning about CAHC in HIV/AIDS and had access to the Internet. This limited recruitment approach was used as this investigation was intended to be a pilot study. A web site address was provided, which linked to more information including details about remuneration and technical requirements for using the ePresence software. Those interested in participating were requested to contact the principal investigator by email. This research protocol was approved by the University of Toronto HIV/AIDS Research Ethical Review Board.

A total of fourteen potential participants that contacted the principal investigator were emailed a brief survey, which asked whether they were able to access a demonstration version of ePresence and also various demographic questions. Two reported technical problems with their computers or Internet connections, which prevented viewing of the ePresence demonstration. Two others who provided telephone numbers were left voice messages when the survey went unanswered. These four individuals were therefore excluded from participating. Two others were sent the screening survey twice and did not respond. They also did not participate. A group of eight were initially offered a place in the study. One person did not complete the online consent form. Emails to this respondent went unanswered. Another completed the online consent form but subsequent emails containing a username and password to access the study environment bounced. The remaining six participated in the study until completion. All communications with these six participants up to the group meeting were by email. Participants were directed to view the video workshop. Access to the environment was provided from March 29^th^, 2004 until the group interview meeting on Tuesday April 20^th^, 2004.

As the study was exploratory in nature the qualitative approach of a group interview was used. All six attended this group meeting and were required to answer a survey about their background in using the Internet, including their participation in mailing lists, message forums, or newsgroups and their use of the study environment. The meeting was facilitated by the author of this study, who has a background in psycho-social research and program evaluation, including focus groups. The facilitator also has a graduate degree in measurement and evaluation. Instructions on how the meeting was to be conducted were read from a script. These included that there were no right or wrong answers and that all ideas, comments, and suggestions were welcome. Group interview questions focused on the participants' general impressions of this study environment and how viewing the video and slide presentations and posting messages aided their learning. Questions about the accompanying web site were also asked. Various prompts and follow-up questions were also employed. The group meeting lasted two hours and was audio-recorded and transcribed by the principal investigator. Participants were paid $75 CAD for their efforts.

Integrity of data entry from the surveys was checked by another researcher. Inter-coder reliability was calculated at 80%. Transcripts were coded using QSR N6. The initial coding employed Wolcott's [19] method of "groups in interaction". Specific comments were categorized based on the web site, the presenter, and the ePresence interface. Other comments were provided about using the Internet. Some material was shared about using anecdotal information in relation to treatment, credibility in content, and learning preferences. After the initial coding, broad categories were identified using Wolcott's [19] analysis technique of searching for patterns in the participants' responses. These categories were referred to as 'positive feedback', 'negative feedback' and 'reflective comments'. More details about the categories are presented in the results section.

## Results

### Survey results

All six participants were male with incomes below $30,000 and were between the ages of 31 to 50. Two had completed high school and the remaining four were post secondary graduates. Five were Caucasian and one identified himself as South Asian. Their length of involvement with the HIV/AIDS community ranged from one and half years to twenty three years. Four participants ranked themselves as experts in using both the Internet and CMC, two as intermediates. All participants had subscribed to a mailing list, newsgroup, or message forum before and all but one had previously posted a message. Five participants viewed the video in the workshop at least once, only one did not watch it to the end. For the question that asked participants about viewing the slides, three choices were provided: whether they noticed the slides at all, somewhat, or considerably. Five stated noticed the slides considerably, one somewhat. Half of the participants stated they had reviewed the slides. Most read all the forum messages, only one did not read any. Also, one did not view the accompanying web site.

### Message postings

The posting of questions or comments was optional but participation was encouraged by providing an introductory message asking the respondents to do so. The principal investigator posted this introductory message in the forum at the beginning of the study. A total of 18 messages were posted from March 29^th ^to April 17^th^, 2004. None directly related to the material presented in the workshop. No messages contained questions for the expert workshop presenter. Many posts were technical questions about ePresence; some were about learning in general and two others included comments about living with HIV. Of the messages posted by the participants, several contained comments about usability of the study environment. About half of the messages posted were responses made by the principal investigator to queries from participants, including those related to functioning of the environment.

### Group interview results

In the group interview a variety of questions about the ePresence interface were asked. These questions started with a general focus about impressions related to the video presentation, slides, forum, and accompanying web site. Subsequent questions focused specifically on each area and how the component impacted on learning. From the responses provided by the participants several sub-themes were labelled during the coding of the transcripts. A large number of comments, especially at the beginning of the group interview, were quite negative in nature. These categories were reviewed by another researcher and considered exhaustive, exclusive, and sensitising [20]. Negative themes ranged from comments about the accompanying web site (e.g., structure not well organized, too much information), ePresence (e.g., slide format not useful, video not well done), the Internet (technical problems with video software, connection issues), to techniques used by the workshop presenter and issues about posting messages online.

Positive comments followed similar sub-themes, except there were no comments shared about message posting or the accompanying web site. For the comments referred to as "reflective" participants shared thoughts about preferences for information formats (written material, audio, and integration of formats), the notion of reading and posting messages (CMC), and meeting information needs in general. Some comments were proprietary to the web site and the workshop presenter used in this study. As such, this material is more relevant to those involved with the development of those components. The deeper analysis material that follows next focused on the three sub-themes from the reflective comments. Some positive and negative comments about the ePresence interface were also provided. Participants were assigned code names, ranging from P11 to P16 to represent the six people at the group interview.

### Reflective thoughts: Preferences for information formats

The following sections provide various quotes from study participants. Each of these quotes was chosen as examples of the theme they represent. In as many cases as possible a theme was illustrated with all the associated comments from participants. Also, material is provided in thick description format to provide the greatest amount of detail.

Individual learning preference was one theme shared by some participants. P13 stated, ... *if I'm given a choice of an audio or print material or a video, I'm always, always, going to chose the print material, because I just want to get my information...so I don't know if that is just a personal preference but I really do like the printed word than anything else*." Subsequent to this comment P16 then stated, "*It's a personal thing because I prefer the audio myself rather than print*." One participant [P14] preferred the combination stating, *"I like the integration." *and P15 agreed. P14 later went on to say, "*So I was really excited that there was a new mode of information dissemination being presented rather than these really thick journals where you scroll through pages and pages of text which I hate doing. Um because I really enjoy the multimedia aspect of things*." These comments demonstrate a variety of preferences for learning. Of the four participants who shared comments, one preferred print, another audio, and two liked the integration of multi-media.

### Reflective thoughts: using CMC

When participants were asked about their views and practices regarding the use of CMC, responses were varied, from having no interest in using the technology to finding it quite useful. P16 stated that he preferred to have experts make decisions for him and did not post messages online concerning his HIV/AIDS health interests. P11 did not consider posting messages for any purpose.

P12 shared that he was not trusting of online posted messages, *"I won't necessarily post a question on a bulletin board you know, not knowing anything, even literally usually the real name or the person who is responding"*. P13 used message forums frequently for other interests. When discussing the issue of trust in online messages he said, *"But also you have to have a large enough number of participants in a bulletin board and somebody says something that's wrong or misleading, it's going to get corrected." *P13 also stated, " *...so this idea of getting help from the community whatever community, isn't strange to me, but I go onto JAVA lang help to get technical support... to whatever problem I'm trying to solve*". When discussing use of message forums as information sources, P14 shared these thoughts: "...*I don't want to sit in a room with a doctor with a lot of credentials listening to them talking about a lot of tables, and charts and numbers, this is absolutely what I do not want to do...I want to sit around and listen to a bunch of people who are HIV positive, who have experience with HIV/AIDS share their personal experience and that's what I, that's how I envision the context of logging into forums as a community thing, rather than an academic journal type of thing." *In summary, some participants did not use CMC at all and one did not trust it as a source. The other three shared that they used this form of communication but not usually to learn about HIV/AIDS treatment information.

### Reflective thoughts: Meeting information needs

In response to statement about asking others for help in understanding treatment information provided by P12, further clarification was requested. He responded, "*Yes, both online and organizations and also medical professionals and also some peers that I know have, have really good connections and ah, their own connections and their own information. So it works like that*." P15 then commented about how he shares information with P14, who is also a friend: "...*Like if I find something that's really relevant to what's going on to him, I can send him a link...this is something worthwhile because P14 knows me... I don't want to read about all this shit and I don't want to get thrown all over the place, this is one specific article about something that is important to me"*. P14 described his own situation as follows, *"...so what I've noticed is that I'm really want to get connected to other people in order to access information, that personal connection, whether it is with a doctor or someone in my support group or some other kind of social service provider or whatever, that is critical for me."*

P16 did not use the Internet as an information source, preferring instead to have experts make any treatment decisions about his care. He shared that he would, "*Let an expert determine what was best for me, based on the state I was in at the time kind of thing*." P11 stated he used the Internet like a reference system, checking content against information he had found elsewhere. To summarize, three participants described ways in which their treatment information needs were being met, including offline sources and communicating directly with each other. Two participants did not use CMC. The final participant did not comment during this discussion.

### Comments about the ePresence environment

A few comments were shared about using the ePresence interface. One that was positive in nature was also very general. P15 stated, "*I like the ePresence environment*." Another more specific comment from P12 included, "*...where things were in the contents, where he was, where the slide was...that tool was a great tool*." This participant was referring to the interactive mechanism found in ePresence that was described above. P15 then added, "*Ya, lining the text up with bar on the bottom is a good idea..*.." Another participant, P13, shared more mixed views. He stated, "*I think the sky's the limit. And I think it is a wonderful idea. It's just not there yet*." Other comments included such statements from P12 as, "*And when along the bottom of my screen there was plenty of room for scrolling text, you know, if that is something they could incorporate into it, where literally the text could scroll up as he is going through it is another means which would actually make the site available to the hearing impaired, among other things*". P13 had some issues with the video component of ePresence. He stated, "*All the video stuff is great but it seems like a lot more trouble than it's worth*."

## Discussion

### Usability issues

A commonly reported problem with the application of web-based computer interfaces, in general, is poor usability. Usability is a computer science concept intended to describe the efficiency (can the interface be used in a timely manner?), effectiveness (can it be used without the user committing errors?), and satisfaction (is it easy and enjoyable to use?) of software applications. The research participants in this study noticed (and were affected by) many of these usability concerns, as briefly outlined in the Group interview results section. Many using the Internet are very savvy about visual media, having already watched copious amounts of television and motion pictures. With few exceptions most have seen countless hours of video in which production quality is very high. Further, many have read large amounts of text-based information, which has been professionally edited and type-set. As a result, some participants in this study may have been negatively impacted components containing imperfections. As one participant (P12) said, "...*that's a whole thing about the Internet, it needs a good edit*". An interface must be very "clean" before effects or impact of learning on participants can be measured for effect on learning or health improvements [21]. It is purposed that this issue is severely hampering progress of this field in general. The problem is also evident in the postings found in the message forum in which concerns over the usability of the study environment were shared. Although learning was not demonstrated through use of the message forum, participants did use it to express their problems with the interface.

### Overall study results

The participants who reported using combined off and online practices to meet information needs in relation to HIV/AIDS were those who had been involved in the community for the longest period of time (9 to 23 years). For these "old timers" a support system or networks for helping them find and process treatment information already existed. As a result, there may be less desire to use technology to learn and especially to discuss HIV content using CMC. For the one participant new to the community (1.5 years), there was more of a reliance on in-person contact. The remaining participant (7 years) was somewhat disconnected from both off and online sources, but did state he preferred to learn information on his own or with his partner. Others in the study reported much longer involvement with HIV/AIDS issues. One participant who had been involved for 9 years did not share details about his use of Internet technologies in relation to HIV treatment, but did speak at length about his use of the resource to learn about computer programming. In reference to using HIV online resources, P13 stated, "I mean I got quickly involved with the HIV community so I didn't really need it." Participation in the community may partially determine in whether someone becomes involved online.

With half the participants clearly expressing no interest in posting messages in an online environment it is difficult to imagine how the message forum in ePresence could be successfully utilized. Further, with the knowledge that many were discussing information with others in an offline context it became apparent that information needs are being met in ways without CMC. Use of the technology appears to be, in some cases, an adjunct to other offline resources. The means and extent to which HIV/AIDS community members meet their information needs is more complex and rich than originally understood. Current need for HIV/AIDS treatment information may play a role in utilization of any information source. Recruiting study participants from online environments would not likely have had any effect. Rather, it is more likely that where they reside and the number of in-person resources that is impacting their need to participate in online forums. It seems as though those from communities such as Toronto, which offer many services for PHAs, may have a less of a need for online sources of information than others.

Based on these findings, this study might be considered a failure. Even after using secure login procedures that ensure complete safety in the ePresence environment and, in particular, the message forum was not well utilized by the participants. It is unclear whether the reluctance to use the message forum was an issue related to the ePresence environment or other issues with computer mediated communication formats. In contradiction with the literature cited stating that HIV/AIDS learners have an interest in complimentary and alternative healthcare and that this population have used computer mediated communication, there was little effort to learn about the content presented in this study's online workshop using CMC. Perhaps the participants did not have a strong enough desire to understand the material and therefore they did not engage with message postings. Regardless of the reasons, the study results were not expected. Some limitations that may have affected this study are explored next.

### Study limitations

The original intention of the study was to conduct a series of focus groups within a grounded theory framework, but as noted, the initial results indicated the study environment was not used by the participants. Therefore, the study was halted and the data collected was limited to six participants. As a pilot study the results were not intended to generalize to the overall population, but rather inform future research. A subsequent study (in preparation) focusing on how information needs are being met was instead undertaken.

The recruitment notices were placed in downtown Toronto, drawing in participants only from this area. Toronto is a large metropolitan city in Canada, with many AIDS Service Organizations and also a large HIV/AIDS population. Therefore, as already noted, it may be that sources of information and help in relation to HIV/AIDS are rich and readily available in this city. Two participants were known to each other in the study. Unfortunately there is no way to recruit participants without this occurring. Recruitment notices cannot advice respondents not to tell others of the study nor prevent friends or acquaintances from participating. Avoiding this issue is unlikely in the often close-knit HIV/AIDS community. In addition, there were only male participants. Women may have provided a completely different experience as their connections in the male prevalent HIV/AIDS community may be more limiting. Also, the web-based software application used in this investigation has some technological limitations, which precluded some from participating.

### The importance of reporting pilot study results

The research presented in this paper discussed feedback from six participants. Based on the results of the study, no further participants were recruited. Not only was it clear that they did not want to use this online environment to learn, nor did they rely on other online environments for HIV/AIDS treatment information, despite a previous publication to the contrary as cited in the Introduction. However, important information was shared in this study that could be of value to other researchers considering implementing or testing similar web-based applications for consumer health education, particularly in HIV/AIDS.

The lack of publication of pilot studies or research that fails is a known issue. As stated by van Teijlingen and Hundley [22], "...it is equally important to ensure that lessons learned with respect to the research method are shared, otherwise patients may be subjected to poorly developed tools or money might be wasted because methods of recruitment failed." (p. 36). Further, it also considered an ethical obligation to publish issues experienced while conducting research, including pilot studies [22].

## Conclusion

Applied implications: Participants did not use the online workshop presented in this study effectively. The message area was not used to aid learning of the content. There was no evidence of it supporting group learning. However, despite an initial focus on negative comments, some positive feedback was obtained about using the Internet to support HIV/AIDS information needs. In general, participants either did not want to use the study environment format to learn about treatment issues and or had access to other sources of information. Those who are considering implementing this type of interface to support other consumer health education efforts should be aware of these types of issues before investing in the technology.

Policy implications: Finding ways to utilize the benefits of an online video, message forums, and support documents are essential to continue supporting web-based learning in the HIV/AIDS community. Other PHAs located in smaller communities may not have access to a wide variety of in-person sources of information and consequently may be relying primarily on web technology for treatment information.

Research implications: Replication of this research in other communities in which these offline resources are not as prevalent is therefore warranted. Less advanced technology may also be better suited, despite literature promoting use of video and multi-media applications. A simple mailing list may be sufficient to support treatment information needs. The findings in this study also indicated that the HIV/AIDS community has complex ways of meeting its information needs. More research on how the community is collaborating in their learning is therefore necessary.

## Abbreviations

AIDS: Acquired Immune Deficiency Syndrome

BBS: Bulletin Board System

CHESS: Comprehensive Health Enhancement Support System

CMC: Computer mediated communication

HIV: human immunodeficiency

PHAs: People with HIV/AIDS

## Competing interests

The author(s) declare that they have no competing interests.

## Authors' contributions

Laura O'Grady is the sole author of this paper. She conducted the literature review, conceptualized the study, determined the research methods, obtained the informed consents, conducted the focus groups, administered the surveys, transcribed the focus group meetings, analyzed the data, and wrote the final paper.

## Pre-publication history

The pre-publication history for this paper can be accessed here:



## References

[B1] Boberg EW, Gustafson DH, Hawkins RP, Chan C, Bricker E, Pingree S, Berhe H (1995). Development, acceptance, and use patterns of a computer-based education and social support system for people living with AIDS/HIV infection. Computers in Human Behavior.

[B2] Kalichman SC, Benotsch EG, Weinhardt L, Austin J, Luke W, Cherry C (2003). Health-related Internet use, coping, social support, and health indicators in people living with HIV/AIDS: preliminary results from a community survey. Health Psychol.

[B3] Gomez EJ, Caceres C, Lopez D, Del Pozo F (2002). A web-based self-monitoring system for people living with HIV/AIDS. Comput Methods Programs Biomed.

[B4] Sharf BF (1997). Communicating breast cancer on-line: support and empowerment on the Internet. Women Health.

[B5] Klemm P, Nolan MT (1998). Internet cancer support groups: legal and ethical issues for nurse researchers. Oncol Nurs Forum.

[B6] Reeves PM (2001). How individuals coping with HIV/AIDS use the Internet. Health Educ Res.

[B7] Gustafson DH, Bosworth K, Hawkins RP, Boberg EW, Bricker E (1992). CHESS: a computer-based system for providing information, referrals, decision support and social support to people facing medical and other health-related crises. Proc Annu Symp Comput Appl Med Care.

[B8] Gustafson DH, Hawkins R, Boberg E, Pingree S, Serlin RE, Graziano F, Chan CL (1999). Impact of a patient-centered, computer-based health information/support system. Am J Prev Med.

[B9] Klemm P, Hardie T (2002). Depression in Internet and face-to-face cancer support groups: a pilot study. Oncol Nurs Forum.

[B10] Robinson TN, Patrick K, Eng TR, Gustafson D (1998). An evidence-based approach to interactive health communication: a challenge to medicine in the information age. Science Panel on Interactive Communication and Health. Journal of the American Medical Association.

[B11] Rice RE, Rice RE and Katz JE (2001). The Internet and health communication. The Internet and health communication: experiences and expectations.

[B12] Gomella LG, Albertsen PC, Benson MC, Forman JD, Soloway MS (2000). The use of video-based patient education for shared decision-making in the treatment of prostate cancer. Semin Urol Oncol.

[B13] Gagliano ME (1988). A literature review on the efficacy of video in patient education. J Med Educ.

[B14] Schotte C, Maes M, Beuten T, Vandenbossche B, Cosyns P, Van Coppenolle F (1993). A videotape as introduction for cognitive behavioral therapy with depressed inpatients. Psychol Rep.

[B15] Staupe A, Hernes MS (2000). How to create a learning environment on the Internet, based on constructivism and sociocultural approaches?.

[B16] Garrison DR, Anderson T (2003). E-learning in the 21st century : a framework for research and practice.

[B17] Duggan J, Peterson WS, Schutz M, Khuder S, Charkraborty J (2001). Use of complementary and alternative therapies in HIV-infected patients. AIDS Patient Care STDS.

[B18] Morgan DL, Krueger RA, King JA (1998). Focus group kit.

[B19] Wolcott HF (1994). Transforming qualitative data : description, analysis, and interpretation.

[B20] Merriam SB (1998). Qualitative research and case study applications in education.

[B21] Badenoch D, Tomlin A (2004). How electronic communication is changing health care: Usability is main barrier to effective electronic information systems. BMJ.

[B22] van Teijlingen E, Hundley V (2002). The importance of pilot studies. Nurs Stand.

